# Reception and Distribution of Cases in a Casualty Clearing Station

**Published:** 1919-01

**Authors:** 


**Reception and Distribution of Cases in a Casualty Clearing Station.** By Captain E. W. N. WOOLER. *American Journal of the Medical Sciences*, August, 1918.

*Reception of Cases.* On arrival, after names have been entered in admission and discharge books, the patients are sent to the dressing rooms, one for lying, and one for sitting cases.

*Dressing Room.* Arrangements should be provided for dressing 8 lying cases at a time; these are arranged in two rows of four, each of the M. O.'s in charge dealing with the cases in one row.

*Annex.* It proves convenient to have part of the dressing room screened off, so as to form an annex where slight operations can be performed under gas or ethyl chloride anesthesia.

*Distribution from Dressing Rooms.* The following cases need to be detained: abdominal cases in which penetration is suspected, and chest cases in which penetration is evident; head cases, when there is evidence of injury to the brain; suspected gas infection wounds; compound fractures of the femur; cases of deep-seated hemorrhage, even if the injured vessel is not a large one; cases suffering from external hemorrhage and shock; and flesh wounds with considerable destruction of muscles.

*Disposal of Cases.* According to the condition of the patient, the cases are either sent immediately to the pre-operation room, for preparation, or to the special wards set apart-for certain wounds, or to the resuscitation ward.

*General Principles Guiding the M. O. in Deciding Whether to Evacuate or Detain a Case.* Bad looking wounds surrounded by tension, edema or inflammation, must be detained. If gas infection is suspected, or if the wound presents a thin brownish discharge exuding under pressure, or an odor "fecal" in character, evacuation should be delayed. Patients who have suffered for several days from exposure to bad weather and starvation should be detained, even if not very severely wounded.

All cases presenting dressings soaked with blood, unsuitable splints, or considerable swelling, indicating some possibility of "concealed" hemorrhage, should be immediately cared for in the dressing rooms; also all cases of gunshot wounds of the head, and those in which the patient complains of much pain, possibly indicating some gas infection.

*Scheme of Labeling.* The labeling must be simple and clear in order to avoid confusion during a great rush. A large red label bearing the letter "E" indicates all cases fit for evacuation. By combining the name of a ward with a special color, there should be no mistake on the part of the stretcher bearers as to the allocation of all cases.

*The Preoperation Room.* Most cases are simply brought into this room before being carried into the theater. This arrangement spares the wards a great deal of work. In addition the patients benefit by remaining on the stretchers until they are operated, and do not have to be lifted from stretcher to bed and vice versa.

*Some Impressions Gathered in the Dressing Room.* In general the wounds are dressed too often, perhaps four times before the men reach the casualty clearing station. That means a considerable waste of time and dressing material, and more harm than good to the wounds. At main dressing stations all lighter wounds should be dressed in such a way as not to need any new dressing until the base hospital is reached, and such cases should be labeled carefully, so that no confusion might arise.

*The Arrangement of Work When Two or More Casualty Clearing Stations are Working in Conjunction.*

(1) Each may be on duty for 24 hours at a time.

(2) Each may receive a definite number of cases (200 for instance).

(3) Each may receive a definite number of cars alternately (6 for instance).

This last scheme is considered by the author as the best one for the patient. The fact that a small number of cars is taken at a turn makes possible dividing the work equally between both stations, and reduces the danger of gas gangrene developing through delay in treatment.

